# Taxonomic and Functional Diversity of Rhizosphere Microbiome Recruited From Compost Synergistically Determined by Plant Species and Compost

**DOI:** 10.3389/fmicb.2021.798476

**Published:** 2022-01-13

**Authors:** Ning Wang, Huixiu Li, Bo Wang, Jia Ding, Yingjie Liu, Yuquan Wei, Ji Li, Guo-Chun Ding

**Affiliations:** ^1^Beijing Key Laboratory of Biodiversity and Organic Farming, College of Resources and Environmental Science, China Agricultural University, Beijing, China; ^2^Organic Recycling Institute (Suzhou) of China Agricultural University, Suzhou, China; ^3^Tangshan Normal University, Tangshan, China

**Keywords:** shotgun metagenomics, 16S *rRNA*, rhizosphere, compost, functional diversity

## Abstract

Compost is frequently served as the first reservoir for plants to recruit rhizosphere microbiome when used as growing substrate in the seedling nursery. In the present study, recruitment of rhizosphere microbiome from two composts by tomato, pepper, or maize was addressed by shotgun metagenomics and 16S *rRNA* amplicon sequencing. The 16S *rRNA* amplicon sequencing analysis showed that 41% of variation in the rhizosphere bacterial community was explained by compost, in contrast to 23% by plant species. Proteobacterial genera were commonly recruited by all three plant species with specific selections for *Ralstonia* by tomato and *Enterobacteria* by maize. These findings were confirmed by analysis of 16S *rRNA* retrieved from the shotgun metagenomics library. Approximately 70% of functional gene clusters differed more than sevenfold in abundance between rhizosphere and compost. Functional groups associated with the sensing and up-taking of C3 and C4 carboxylic acids, amino acids, monosaccharide, production of antimicrobial substances, and antibiotic resistance were over-represented in the rhizosphere. In summary, compost and plant species synergistically shaped the composition of the rhizosphere microbiome and selected for functional traits associated with the competition on root exudates.

## Introduction

Organic waste from intensive animal farms and agriculture is a massive reservoir of nutrient elements. For example, the animal manure produced annually in China contains as much as 78 million tons of NPK, which were 10 million tons more than the consumption of plant nutrients (Bai et al., 2016; Hu et al., 2017; Shen et al., 2018). Organic waste recycling can effectively reduce the use of chemical fertilizers and environmental pollution. Composting is a convenient method for recycling organic waste and it is widely applied globally (Wang et al., 2020). Frequently, compost is used as organic fertilizer to replenish nutrients, organic matters to arable soil, or as growing substrate. Several studies have demonstrated that compost fertilization can improve not only soil fertility (Ding et al., 2019; Singh et al., 2020; Wu et al., 2020) but also several ecological services in agroecosystems including mitigation of nitrate leaching (Kramer et al., 2006; Han et al., 2017; Picariello et al., 2020) and maintenance of plant health (Kalbani et al., 2016). It has been demonstrated that compost is suppressive to several soil-borne diseases such as *Fusarium*, *Verticillium*, or *Phytophthora* (Lang et al., 2012; Wu et al., 2017; Bellini et al., 2020). Beneficial microorganisms in composts may contribute to the suppressiveness as proved largely by two lineages of evidence: (1) several bacteria (*Pseudomonas*, *Bacillus*, *Enterobacter*) or fungi (*Trichoderma*, *Gliocladium*, *Penicillium*) in compost are antagonists against pathogens causing soil-borne diseases (Mehta et al., 2014); (2) the ability of compost to suppress plant diseases was often reduced significantly or even lost after sterilization (Hadar, 2011).

Rhizosphere, a narrow region around plant roots, harbors an enormous diversity of microorganisms that may contribute to the well-being of crops. Engineering rhizosphere microbiome is proposed as an avenue to sustainable and productive agroecosystems (Ahkami et al., 2017). Previously, several studies have demonstrated that biotic and abiotic factors, such as soil type, plant species and development, and fertilization regimes, played essential roles in the recruitment of rhizosphere microbiome (George et al., 2017; Xu et al., 2017). Rhizosphere bacteria may provide first line of defense against pathogens causing soil-borne diseases via several mechanisms such as producing antimicrobial substances (Sulistyani et al., 2021), competing for niches (Bauer et al., 2018), triggering plant immune systems (Teixeira et al., 2019), mineralizing of organic materials (Richard et al., 2017), solubilizing recalcitrant phosphorus, or nitrogen fixation (Alori et al., 2017).

Compost fertilization can drive the assembly of the rhizosphere microbiome in several crops, such as maize (Qiao et al., 2019), cucumber (Huang et al., 2017), pepper (Li et al., 2019a), and rice (Liu et al., 2021), and such alternation may attribute to soil physicochemical changes by compost (Liang et al., 2018). In seedling nursery, compost is frequently used as growing substrate, which may provide first reservoir for plant to recruit its microbiome (Anastasis et al., 2017). Recently, it was shown that the microbiome initially colonized in the rhizosphere of tomato predetermined the survive of plant from bacterial wilt disease (Wei et al., 2019). The succession of microbial community is very dynamic during composting. The thermophilic stage of composting (> 55°C) often lasts for more than 5 days, serving as a habitat filter to select for thermophilic bacteria, but against several mesophilic bacteria (Meng et al., 2019). Although some mesophilic microorganisms may re-grow at the maturation stage (Antunes et al., 2016), the composition of the bacterial community in compost significantly differed from those in soil, with a dominance of phyla such as Firmicutes and Actinobacteria (Cerda et al., 2018). Recently, a survey of microbiome in 116 compost samples collected from 16 provinces in China revealed that none of the 26 OTUs in the compost core microbiome are associated with plant disease suppression (Wang et al., 2020). Proteobacterial and Actinobacterial isolates from a disease suppressive compost were still able to proliferate in the rhizosphere of tomato (Anastasis et al., 2017). In general, it is still not well understood which taxonomic and functional diversity could be recruited into plant rhizosphere from compost.

In the present study, the shotgun metagenomics and 16S *rRNA* amplicon sequencing were applied simultaneously to examine the taxonomic and functional diversity recruited by pepper (*Capsicum annuum* L.), tomato (*Solanum lycopersicum* L.), and maize (*Zea mays* L.) from two composts. The acquired knowledge may deepen our understanding on microbial ecology in the rhizosphere.

## Materials and Methods

### Compost

Two composts were made from cow manure (DZ) or a mixture of chicken and cow manure (QZ), respectively. The aerobic fermentation was performed in a window for 25–30 days with a thermophilic period (> 55°C) over 5 days. The nitrogen and organic matter contents were measured according to the methods described in the Chinese national standards (NY525-2012). The 1:10 (w/v) water suspension of compost was used to measure pH with a pH meter (PHS-3C, China). Phosphorus and potassium were analyzed according to standard protocols. The physicochemical properties of composts are given in Table 1.

**TABLE 1:** Physicochemical properties of cow manure compost (DZ) and chicken and cow co-compost (QZ).

Parameter	QZ	DZ
Total nitrogen (%)	2.93 ± 0.60	0.98 ± 0.016
Organic matter (%)	44.41 ± 6.89	25.52 ± 0.398
pH	7.46 ± 0.25	8.15 ± 0.055
Available phosphorus (g kg^–1^)	1.62 ± 0.08	1.89 ± 0.051
Available potassium (g kg^–1^)	5.66 ± 0.08	1.90 ± 0.040

### The Experimental Setup

The experiment was set up as follows: pepper (*C. annuum* L. Zhongliangxin), tomato (*S. lycopersicum*, Baiguofengqiang), and maize (*Zea mays* L. Nongda86) seeds were sown in pots (12 cm in diameter and 15 cm in depth) filled with 600 g of sterilized sand and 120 g of composts. After germination, the seedling was cultivated for 40 days in a climate chamber (Hangzhou Lvbo Instrument Co., Ltd LB-1000D-LED) at 30°C, 70% relative humidity, and 12-h light (15,000 l×) period. Each treatment contained three independent replicates from three pots. Rhizosphere samples were taken as previously described and the pellet was kept at –20°C for DNA extraction.

### TC-DNA Extraction, 16S *rRNA* Amplicon Sequencing, and Shotgun Metagenomics

Total microbial community DNA was extracted using a FastDNA spin Kit for soil (MP Biomedicals, Santa Ana, Carlsbad, CA, United States). The 16S *rRNA* gene fragments were amplified with the universal primer 515F (5′-GTGCCAGCMGCCGCGGTAA-3′) and 806R (5′-GGACTACVSGGGTATCTAAT-3′) fused with a 12-bp unique barcode (Lucas et al., 2015). The PCR products were gel-purified, and equal molar quantities for each sample were mixed for high-throughput sequencing using the platform of Illumina NovaSeq PE250. An equal amount of total microbial community DNA from three replicates of each treatment was pooled for shotgun metagenomics analysis. Library preparation and sequencing were performed according to the standard protocol of Illumina company. The data presented in the study are deposited in the NCBI SRA with the BioProject number PRJNA789701.

### Bioinformatics Analysis

The analysis of 16S *rRNA* amplicons sequencing was performed according to previous descriptions (Wang et al., 2020). Briefly, sequences without ambiguous base “N” and a length > 200 bp were assigned to different samples based on barcodes and primer sequences, which were also trimmed out. Chimera sequences were removed cooperatively using the ChimeraSlayer and a standalone BLASTN analysis against the SILVA database (version 138). Sequences were grouped into different operational taxonomic units (OTUs) (> 97% sequence identity) using the software Vsearch (Rognes et al., 2016). Representative sequences of each OTU were classified using the RDP MultiClassifier at > 50% confidence. Alpha-diversity indices, principal coordinates analysis, identification of discriminative genera, and variation partition were performed according to Li et al. (2019a).

Fragments of 16S *rRNA* sequences were extracted from the shotgun metagenomic library using Bwotie2, and the retrieved sequence was further validated by a standalone BLASTN against the SILVA database (version 138). Classification of the retrieved 16S *rRNA* was also performed with RDP MultiClassifier. Based on the classification, a taxonomic reporter was created with each row represent taxa and the read numbers for each sample. To analyze the functional diversity, sequences were mapped to different gene clusters in the Uniref90 database using the software DIAMOND. Gene clusters were assigned to different taxa based on the taxonomy from the National Center for Biotechnology Information (NCBI).^1^ The assignment of genes to ortholog was cross-linked based on Kyoto Encyclopedia of Genes and Genomes (KEGG) databases. A data frame was constructed in which each row represents a gene cluster (uniref90) with its taxonomy and ortholog and the number of reads for each sample. The number of genes was further adjusted by the number of reads in the library and the gene length into the number of reads per kilobase per billion reads (RPKB). The abundance of different functional groups was summarized by mapping the ortholog into the BRITE database from KEGG. Statistical analysis, UPGMA cluster, and heatmap analysis were performed with the software R. These tools have been implemented into a galaxy instance.^2^

## Results

### Rhizosphere Bacterial Community Recruited From Compost Were Synergistically Shaped by Plant Species and Compost as Revealed by 16S *rRNA* PCR Amplicon Sequencing

**FIGURE 1:**
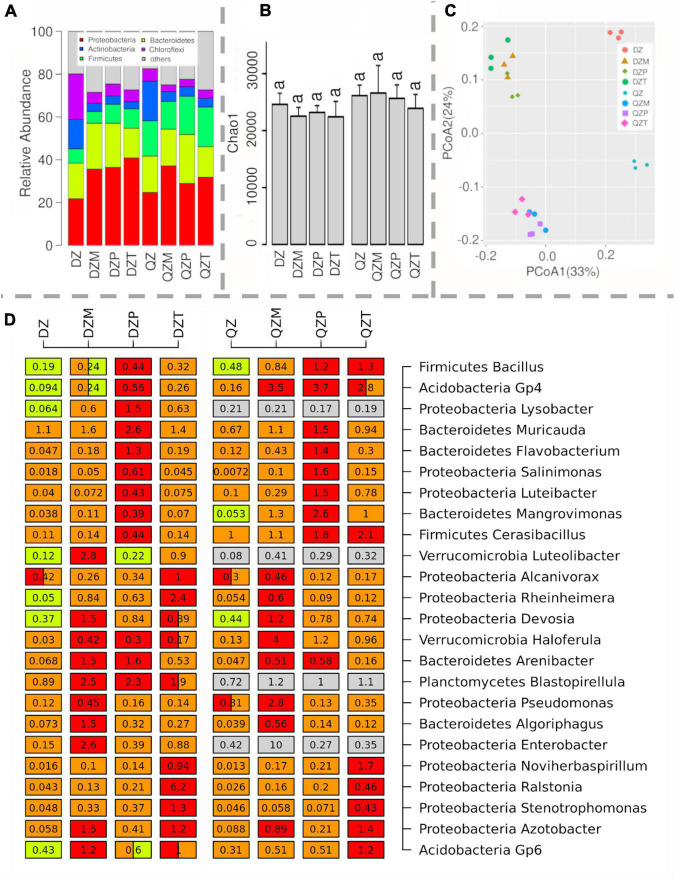
Microbial relative abundance of different phylum **(A)** and their richness **(B)** in the rhizosphere of maize (M), pepper (P), and tomato (T) seedlings cultivated in cow manure compost (DZ) and chicken and cow manure co-compost (QZ) by 16S *rRNA* PCR amplicon sequencing. Principal coordinates analysis (PCoA) showing beta-diversity between rhizosphere and compost or between different composts **(C)**. Genera enriched at different rhizosphere under two compost treatments **(D)**. Numbers on the box indicate relative abundance expressed as a percentage. Significant difference is indicated by a different color. A box with two colors indicates no significant difference from other treatments containing one of the two colors. Significant differences are indicated by different letters.

A total of 24 rhizosphere samples from maize, pepper, and tomato cultivated in two composts were analyzed by Illumina sequencing of PCR-amplified 16S *rRNA* gene fragments. Proteobacteria (21.8–40.8%), Bacteroidetes (13.9–22.9%), Firmicutes (5.4–18.5%), and Actinobacteria (3.3–18.5%) were abundant in the community (Figure 1A). As expected, the relative abundances of Proteobacteria in the rhizosphere were 17.0–87.1% higher than those in composts (Figure 1A). An opposite trend was observed for Actinobacteria, of which relative abundances were 71.5–77.8% lower in the rhizosphere than those in compost (Figure 1A). No effect of compost or plant species on the richness of rhizosphere bacterial communities was observed (Figure 1B). Principal coordinates analysis indicated that bacterial communities were separated between rhizosphere and compost or between different composts (Figure 1C). Permutation analysis revealed that the effects of compost (QZ vs. DZ *d*-value = 17.7%) on rhizosphere bacterial community were stronger than that by different plants (maize vs. tomato: *d*-value = 10.0%, maize vs. pepper *d*-value = 10.2%, and pepper vs. tomato *d*-value = 9.5%). Variation partition analysis confirmed that compost could explain 41% of variation in the rhizosphere bacterial community compared with 23% variation by plant species. These results demonstrated that compost and plant were major factors shaping the rhizosphere bacterial communities recruited from composts.

Abundant genera which were significantly (*p* < 0.05) enriched in the rhizosphere were identified by multiple comparisons. Among them, 12 out of 25 were affiliated with Proteobacteria, followed by Bacteroidetes (*Arenibacter*, *Flavobacterium*, *Mangrovimonas*, *Muricauda*, and *Algoriphagus*), Firmicutes (*Bacillus* and *Cerasibacillus*), Acidobacteria (*Gp4* and *Gp6*), Verrucomicrobia (*Haloferula* and *Luteolibacter*), and Planctomycetes (*Blastopirellula*) (Figure 1D). Interestingly, most genera were explicitly selected by different plant species (Figure 1D). For example, *Muricauda*, *Flavobacteria*, *Salinimonas*, and *Luteibacteria* were only significantly enriched in the rhizosphere of pepper (Figure 1D). *Noviherbaspirillum*, *Ralstonia*, and *Stenotrophomonas* were enriched in the rhizosphere of tomato. *Algoriphague* and *Enterobacteria* tended to be enriched in the rhizosphere of maize (Figure 1D). In contrast, *Devosia* were commonly enriched in the rhizosphere of all plants grown in two different composts (Figure 1D). In addition, *Bacillus* and *Acidobacteria Gp4* were also enriched in all rhizosphere samples except for the maize grown in the cow manure compost (Figure 1D). *Lysobacter* was only enriched in the rhizosphere of plants grown in the compost from cow manure (Figure 1D).

### Effects of Compost and Plant Species on the Rhizosphere Bacterial Community Were Partially Confirmed by the Analysis of 16S *rRNA* Sequences Retrieved From the Metagenomic Library

**FIGURE 2:**
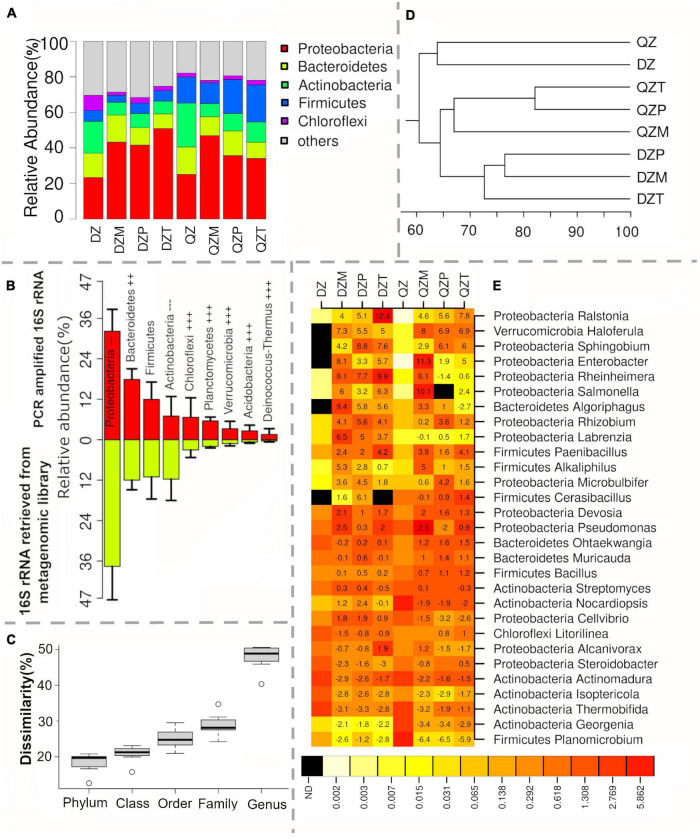
Microbial community by the 16S *rRNA* sequences retrieved from shotgun metagenomics revealed relative abundance of different phylum in the rhizosphere of maize (M), pepper (P), and tomato (T) seedlings cultivated in cow manure compost (DZ) and chicken and cow manure co-compost (QZ) **(A)**, the discrepancy of 16S *rRNA* PCR amplicon sequencing on dominant phylum **(B)** and community composition at different taxonomic level **(C)**, separation on community composition by the UPGMA clustering analysis **(D)**, and dominant enriched genera in the rhizosphere. Numbers on the box indicate the level of enrichment by log2 of relative abundance of each taxon between rhizosphere and compost **(E)**.

The analysis of 16S *rRNA* sequences retrieved from the metagenomic library again revealed that Proteobacteria (23.4–50.9%), Firmicutes (3.9–20.9%), Bacteroidetes (8.3–15.4%), and Actinobacteria (7.4–24.6%) were the most detected phyla (Figure 2A). However, the relative abundance of Proteobacteria and Actinobacteria by the metagenomic profiling were significantly lower than those by the 16S *rRNA* amplicon sequencing (Figure 2B). In contrast, opposite trends were observed for Bacteroidetes, Chloroflexi, Planctomycetes, Verrucomicrobia, Acidobacteria, and Deinococcus-Thermus (Figure 2B). The dissimilarity in bacterial community composition between two methods increased from 12.6–20.2% at the phylum level to 40.4–50.6% at the genus level (Figure 2C), indicating more discrepancies between the two profiling methods at the finer taxonomic levels. Nevertheless, the UPGMA clustering analysis still confirmed that compost was a major factor shaping bacterial taxonomic composition in the rhizosphere (Figure 2D).

The most dominant five genera in each treatment were selected for further analysis and a total of 29 genera were acquired (Figure 2E). Relative abundance of *Ralstonia* and *Haloferula* were strongly (> 7-fold) elevated in all rhizosphere (Figure 2E). Relative abundance of *Sphingobium*, *Enterobacter*, *Rheinheimera*, *Salmonella*, *Rhizobium*, *Labrenzia*, and *Algoriphagus* were strongly (> 7-fold) increased in the rhizosphere of plants grown in the DZ compost (Figure 2E). These genera were only occasionally enriched at a large magnitude in the rhizosphere of plants grown in the QZ compost (Figure 2E). Again, *Ralstonia* was most enriched (221-fold in QZ and 5,403-fold in DZ) in the rhizosphere of tomato (Figure 2E). Also, the strongest enrichment of *Enterobacter* (2,520-folds in QZ and 273 folds in DZ) was also detected in the rhizosphere of maize (Figure 2E). *Devosia* and *Paenibacillus* were also commonly but at a less magnitude (> 1-fold) enriched in all rhizosphere (Figure 2E). *Othaekwangia* and *Muricauda* were slightly (> 1-fold) enriched in all rhizosphere of plants grown in the QZ compost (Figure 2E). *Bacillus* was only slightly enriched in the rhizosphere of pepper of tomato grown in the QZ compost (Figure 2E). *Nocardiopsis* and *Cellvibrio* tended to be enriched in the rhizosphere of plants grown in the DZ compost, but decreased in the QZ compost treatment (Figure 2E). Relative abundances of *Planomicrobium*, *Georgenia*, *Thermobifida*, *Isoptericola*, and *Actinomadura* were 53.3–98.9% lower in the rhizosphere than those in composts (Figure 2E).

### An Utmost Re-assemblage of Functional Diversity in the Rhizosphere

**FIGURE 3:**
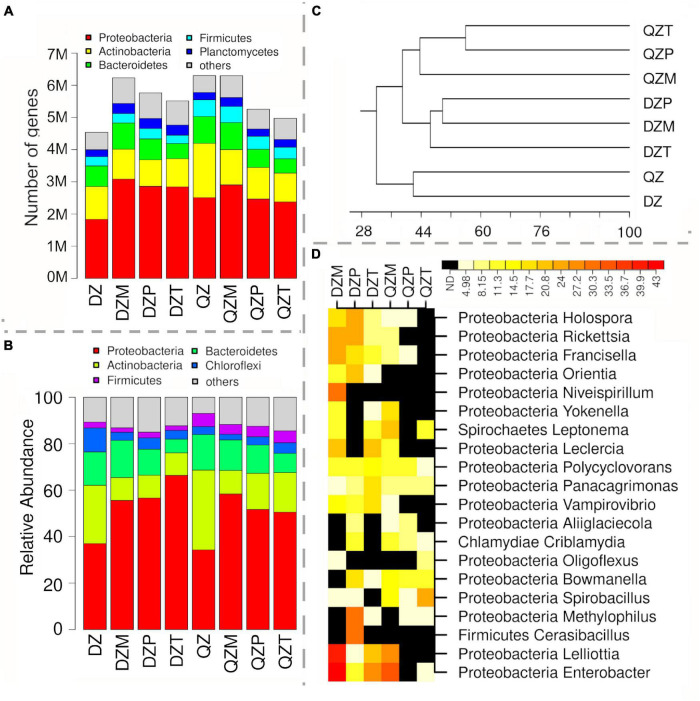
Microbial community as revealed by functional gene clusters using shotgun metagenomics. The amounts expressed in million **(A)** and relative abundance **(B)** of functional gene clusters affiliated to different phyla, difference in functional gene clusters between rhizosphere **(C)** and compost and the top 5 genera most enriched in the rhizosphere as indicated by log2 of fold increase in gene abundance **(D)**.

The diversity of functional genes detected was extremely high for both compost and rhizosphere samples, with a total of 15,206,729 different gene clusters. For each treatment, the number of detected gene clusters ranged between 4.54 and 6.30 million (M) (Figure 3A). The majority of gene clusters were affiliated with Proteobacteria (1.83–3.08 M), Actinobacteria (0.82–1.69 M), Bacteroidetes (0.46–0.84 M), Firmicutes (0.26–0.50 M), and Planctomycetes (0.22–0.31 M) (Figure 3A). The relative abundance of gene clusters affiliated with Proteobacteria was lower in compost than in corresponding rhizosphere samples. It increased from 38.2% in DZ compost to 57.9, 61.6, and 67.8%, 34.1% in QZ compost to 57.9, 54.9, and 52.9% in the rhizosphere of maize, pepper, and tomato, respectively (Figure 3B). An opposite trend was observed for gene clusters affiliated with Actinobacteria (Figure 3B). These results agree with the findings of the two profiling methods. Again, the composition of gene clusters was different between rhizosphere and compost. The similarity on gene cluster composition between compost and rhizosphere samples (25.7–54.2%) was much lower than those (54.8–82.3%) based on profiling methods (Figure 3C). Correspondingly, 67.9–72.4% gene clusters differed more than sevenfold in abundance between rhizosphere and compost, indicating an utmost re-assemblage of rhizosphere microbial communities in terms of functional diversity.

Divergent responses to plant rhizosphere were common among different genera. In general, proteobacterial genera tended to harbor more gene clusters enriched in the rhizosphere, in contrast to genera affiliated to Actinobacteria, Bacteroidetes, or Firmicutes (Supplementary Figure 1). In general, fractions of functional gene clusters within different genera were positively correlated with the magnitudes of enrichment (Supplementary Figure 1). Few genera were high in the fraction of enriched gene clusters but low in the magnitude of enrichment, indicating rare species within these genera were enriched in the rhizosphere (Supplementary Figure 1), while a high magnitude of enrichment but a low fraction of enriched gene cluster indicates that dominant species within the genera were enriched in the rhizosphere (Supplementary Figure 1). The top 5 genera with the highest fraction of gene clusters enriched in the rhizosphere of each treatment were selected for a heatmap analysis (Figure 3D). Both compost and plant synergistically selected genera with the highest fraction of enriched gene cluster. In general, the plant selection was stronger for the DZ compost than the QZ compost, as evidenced by a higher ratio of enriched genes to diminished genes (Figure 3D). Three proteobacterial genera (*Enterobacter*, *Lelliottia*, and *Leclercia*) were among the top 5 genera with the highest fraction of gene cluster enriched in the rhizosphere of maize grown in both composts and tomato grown in the DZ compost (Figure 3D). *Vampirovibrio*, known as bacterivore, was also ranked as the top 5 genera in the rhizosphere of tomato grown in the DZ compost (Figure 3D). The divergent response was detected for *Cerasibacillus* with the highest fraction of gene clusters enriched in the rhizosphere of pepper treated by the DZ compost. Functional genes affiliated to *Ralstonia* were most enriched in the rhizosphere of tomato and functional genes of *Enterobacter* and *Lelliotia* and *Salmonella* were enriched in the rhizosphere of maize. Functional genes associated with *Vampirovibrio* were most enriched in the rhizosphere of pepper treated by the DZ compost (Figure 3D).

### Functional Properties Selected for or Against the Plant Rhizosphere

**FIGURE 4:**
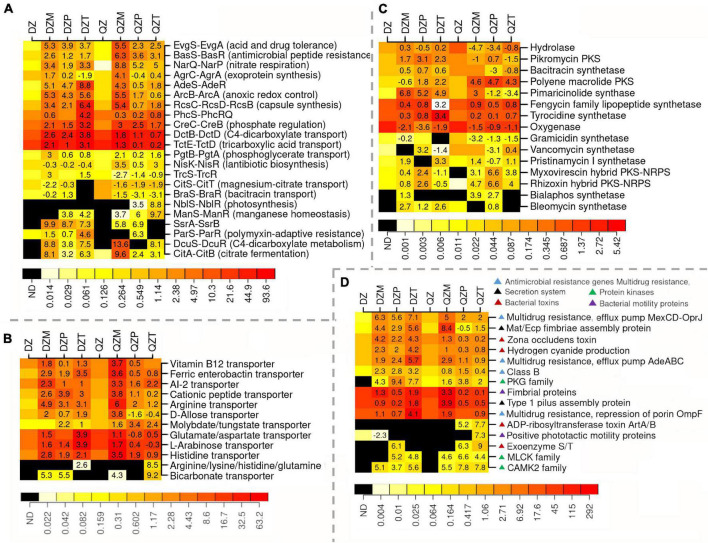
Functional properties selected for and against different plants. Two-component systems **(A)**, transporter **(B)**, polyketide synthesis **(C)**, and other subgroups associated with antimicrobial resistance, production of toxins and protein kinases, motility, and secretion system **(D)**. Different color indicates RPKB values and number in box indicates log2 of the ratios on subgroup abundance between rhizosphere and compost.

To understand the functional properties of rhizosphere microbiome, subgroups within transporters, two component systems, protein kinases, antimicrobial resistance, toxins, polyketide synthesis, motility, and secretion system were compared between rhizosphere and composts (Figure 4). A high abundance of sequences matching two-component systems associated with the transport or the metabolisms of C3 and C4 carboxylic acids (*TctED*, *DctBD*, *DcuSR*, *CitAB*, *DcuSR*), the response to physicochemical or antibiotics stress (*EvgSA*, *BasSR*, *AdeSR*, *ParSR*), the production of pathogenic effectors or antibiotics (*NisKR*, *SsrAB*, *TrcSR*, *PhcSRQ*) or nitrate respiration (*narQP*) were detected in the rhizosphere (Figure 4A). Genes associated with transporters of amino acids (histidine, arginine, lysine or glutamine, glutamate/aspartate), monosaccharide (D-allose, L-arabinose), molybdate/tungstate, siderophore ferric enterobactin, quorum-sensing molecular AI-2, or vitamin B_12_ were also over-represented in the rhizosphere (Figure 4B). The association of genes related to polyketide biosynthesis with rhizosphere largely depended on compost or plant species (Figure 4C). Genes encoding for hydrolase and oxygenase were less representative in the rhizosphere (Figure 4C). Genes related with the biosynthesis of polyene macrolide polyketide, myxovirescin, and rhizoxin hybrid PKS-NRPS were more abundant in the rhizosphere of plants fertilized by the QZ compost (Figure 4C). Genes in the pathway for biosynthesis of pikromycin, pimaricinolide, and bleomycin polyketide were over-represented in the rhizosphere of the plant by DZ compost (Figure 4C). Functional groups associated with antimicrobial resistance, biosynthesis of bacterial toxins and polyketide synthesis, motility, or secretion system tend to be over-represented in the rhizosphere (Figure 4D). Genes related with PKG, MLCK, and CAMK2 family kinase were often more abundant in the rhizosphere (Figure 4D). Genes associated with bacterial toxins such as zona occludens toxin and HCN were more abundant in the rhizosphere of plants by DZ compost, while a high abundance of sequences related with ADP-ribosyltransferase toxin ArtA/B, and Exoenzyme S/T in the rhizosphere of maize and pepper by the QZ compost (Figure 4D). Genes associated with multidrug resistance (efflux pump MexCD-OprJ, AdeABC) were over-representative in the rhizosphere, while there was a high abundance of sequence of Class B Beta-Lactamase in the rhizosphere of plant by DZ compost (Figure 4D). Genes associated with the fimbrial proteins of pilus system also tend to be abundant in the rhizosphere (Figure 4D).

## Discussion

Compost is widely used as organic fertilizer or growing substrate, which may provide the first reservoirs of microbiome recruited by plant seedlings (Anastasis et al., 2017). However, it has been rarely studied the rhizosphere microbial community directly recruited from compost via an advanced metagenomics approach. Herein, both 16S *rRNA* amplicon sequencing and shotgun metagenomics were applied to study the rhizosphere microbiome, and this approach allows us to cross-validate taxonomic changes between rhizosphere and compost. Both metagenomics profiling analyses indicated that microbial composition was clearly separated between compost and rhizosphere. Compost exerted a more substantial effect than those by plant species. These findings are similar to those studies with different soil type, which strongly affects the community of rhizosphere microbiota than the host species (Uroz et al., 2010; Bulgarelli et al., 2012; Lundberg et al., 2012). It is worth noting that rhizosphere microbial communities were only analyzed at the seeding stages in the present study. Rhizosphere microbial community is dynamic over different development stages, and the most substantial effects of plant were often observed at the later stages (Li et al., 2019b). The microbial richness, as indicated by the Chao1 index, was comparable between rhizosphere and composts. It seemed that microbial diversity in the rhizosphere was not a subset of the compost microbiome. It is possibly due to the limitation of Chao1 on measuring the richness of complex microbial communities such as soil, sediment, or rhizosphere, as this index indicated more the distribution of species abundance rather than species richness as the calculated index are related with sequencing depth.

### Bacterial Taxa Recruited in the Rhizosphere and Its Implication

Rhizosphere microbiome provides beneficial effects on the host plant by chemical compounds that can stimulate plant growth and tolerance to stress (Hashem et al., 2019). Both metagenomics profiling indicated that Proteobacteria were enriched in the rhizosphere as observed by others (Carcer et al., 2007; Alzubaidy et al., 2016; Dong et al., 2019). Some of these enriched genera, such as *Pseudomonas* (Silby et al., 2011; Wang et al., 2021) and *Burkholderia* (Xu et al., 2020), served as bioagents. Members of the Bacteroidetes, especially those belonging to the *Flavobacterium* (Figure 1D), were often highly abundant in the rhizosphere of a wide array of plants (Bulgarelli et al., 2012; Bodenhausen et al., 2013; Schlaeppi et al., 2014), and *Flavobacteria* are well known to have been associated with the suppression of *R. solanacearum* (Peterson et al., 2006; Kolton et al., 2014; Kwak et al., 2018). In general, the genus *Bacillus* is prevalent in both soil and plant rhizosphere (Hashem et al., 2019), which were involved in the suppression of several plant diseases (Elshaghabee et al., 2017) via the synthesis of many secondary metabolites, hormones, cell wall–degrading enzymes, and antioxidants (Hashem et al., 2019).

Here, the selection by the plant was likely to be uneven within different proteobacterial genera (Deng et al., 2018). Here, *Ralstonia* and *Enterobacteria* were enriched up to 5,400- and 2,400-fold in the rhizosphere of tomato or maize, respectively. Strong enrichment of *Enterobacteria* in the rhizosphere of maize was also reported in other studies (Mukhtar et al., 2019). *Ralstonia* is known to harbor a pathogen *R. solanacearum*, which caused the most destructive disease in tomatoes worldwide (Wang et al., 2019). It has been demonstrated that *R. solanacearum* could harness several mechanisms, including sensing exudates from tomato roots, and chemotaxis to approach and proliferate in the rhizosphere roots (Wen et al., 2020). Other *Ralstonia* bacteria, which are close relatives of *R. solanacearum*, may also harness similar mechanisms to colonize the rhizosphere of tomato. *Vampirovibrio*, known as a predator of *Cyanobacterium* (Soo et al., 2015), was enriched greatly in the rhizosphere, indicating that the increased abundance of bacteria in the rhizosphere may also attract predator bacteria, some of which can invade cells of gram-negative bacteria. The consumption of some rhizosphere bacteria by predators might also benefit the plant with nutrients as bacterial growth on substrates with low C:N ratio (e.g., bacteria) tend to release additional ammonia or other nutrients. Nevertheless, further studies are still needed to illustrate whether plant do bacteria farming in its rhizosphere.

It is also worth to point out that the discrepancy between two metagenomic profiling methods was also evident, especially under finer taxonomic levels. It has been known that PCR amplification may cause biases as the “universal” primers do not cover all bacteria (Kanagawa, 2003). In addition, degenerate primers can distort the microbial community structure due to inefficient primer–template interactions (Naqib et al., 2019). Thus, changes revealed by PCR-based amplicon sequencing are possibly needed to be cross-validated by other approaches.

### Comparison of Functional Gene Diversity Indicated That Plant Selection in the Rhizosphere Might Be Underestimated by 16S *rRNA* Amplicon Sequencing

It was widely recognized that microbial composition in the rhizosphere was dramatically different from that in bulk soils (Praeg and Illmer, 2020). To our surprise, ca. 70% of functional genes differed at least sevenfold in abundance between rhizosphere and compost, suggesting an utmost re-assemblage of the microbiome in the rhizosphere in terms of functional diversity. The other lineage of evidence that the similarity between compost and rhizosphere was also much lower in functional gene composition than taxonomic composition. These findings agree with studies on comparative genomes, in which the composition of functional genes varied greatly for closely related species (Loper et al., 2012). In agreement with the analysis on the taxonomic composition, genera affiliated with Proteobacteria tend to be more abundant in the rhizosphere (Fierer, 2017). However, divergence in responses to plant rhizosphere might be also common among bacterial species as indicated in Supplementary Figure 1. These findings disagree with the assumption that the rhizosphere microbial community recruited from different environment settings may be varied in taxonomic composition but similar in functions. In contrast, our results indicated an utmost selection in the rhizosphere by plants, highlighting the importance of rare populations.

### Functional Traits Indicated Fierce Competition for Root Exudates Within Microorganisms in the Rhizosphere

Shotgun metagenomics also provides an opportunity to explore the functional diversity recruited by plants (Sharpton, 2014). The acquired knowledge deepened our understanding on the microbial mechanisms associated with the adaptation or competition in plant rhizosphere. Plant regulates the abundance and activities via root exudates, a complex mixture of low molecular weight organic acids, amino acids, or polysaccharides such as mucilage (Canarini et al., 2019). Herein, functional populations related with two-component systems for C3, C4 carboxylic acids and transporter for amino acids and oligosaccharides were more abundant in the rhizosphere. These results suggested that microorganisms with the ability to sense and uptake these substances with small molecular weight might be competent in the rhizosphere. Indeed, wide taxa of rhizosphere bacteria such as *Pseudomonas* and *Bacillus* can sense carboxylic acids and amino acids, which may stimulate bacterial mobility (Taguchi et al., 1997; Alvarez-Ortega and Harwood, 2007; Sampedro et al., 2015; Feng et al., 2021). An increase of functional traits associated with amino acid competition indicated a fascinating relationship between plant and rhizosphere microorganisms in terms of N-exchanging. It has been recognized that legume plants exchange Rhizobia bacteria with small molecular carbons and amino acids for ammonia (Lodwig et al., 2003). However, it is still unknown whether such a mechanism can be extended to the rhizosphere. Rhizosphere, a narrow region containing a large amount of rhizodeposition, is not only a place of banquet but also an arena for microorganisms living there. Microorganisms can employ several types of machinery suppressing others to compete for nutrients and niches (Bauer et al., 2018). Herein, functional populations associated with the biosynthesis of antimicrobial substances such as polyketides, HCN, or toxin were over-representative in the rhizosphere, indicating that abilities to produce antimicrobial substances are also advantageous for surviving in the rhizosphere. The production of HCN or polyketides may also contribute to plant health by suppressing several phytopathogens such as *Fusarium moniliforme*, *F. graminearum*, *P. syringae* pv. *coronafaciens*, *Erwinia carotovora* pv. *carotovora* (Rijavec and Lapanje, 2016), and *Caenorhabditis elegans* (Nandi et al., 2015). Indeed, arms race between antimicrobial substance production and antibiotic resistance in microorganisms possibly lasted for billions of years (Davies and Davies, 2010). Here, functional populations associated with antibiotic resistance machineries, such as multidrug efflux pump and Class B Beta-Lactamase, were enriched in the rhizosphere. These results suggested that some rhizosphere bacteria may harness these antibiotic resistance machinery to overcome constraints of antimicrobial substances exposed by other microorganisms (Davies and Davies, 2010).

**FIGURE 5:**
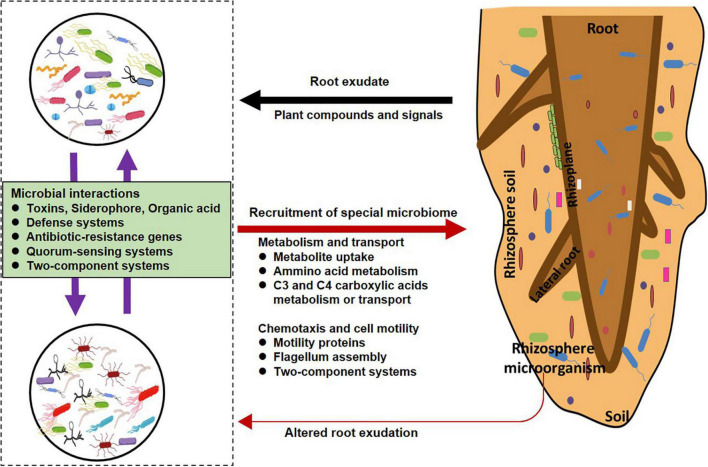
Mechanisms of plant–microbe and microbe–microbe interactions in the rhizosphere. Plants can release root exudates into soil to manipulate soil microbial community assembly; they can recruit and are affected by special microorganisms. Microbial community is also affected by intense microorganism–microorganism interactions mediated via the strain-specific production and quorum-sensing or two-component systems.

Taken together, the metagenomic analysis suggested that the rhizosphere microbiome may employ several mechanisms such as those associated with the sensing and up-taking of root exudates, and production of antimicrobial substance or toxins or resistance antibiotics to enable them to successfully colonize the rhizosphere as illustrated in Figure 5.

## Conclusion

Our results indicated that compost and plant species synergistically determined the recruitment of rhizosphere microbiome with functional properties, which may relate to competition for carboxylic and amino acids.

## Data Availability Statement

The datasets presented in this study can be found in the NCBI SRA with the BioProject number PRJNA789701.

## Author Contributions

G-CD contributed to the conception of the study. NW, HL, BW, JD, and YL performed the experiment. NW and HL contributed significantly to analysis and manuscript preparation. NW and G-CD performed the data analyses and wrote the manuscript. YW and JL helped perform the analysis with constructive discussions. All authors contributed to the article and approved the submitted version.

## Conflict of Interest

The authors declare that the research was conducted in the absence of any commercial or financial relationships that could be construed as a potential conflict of interest.

## Publisher’s Note

All claims expressed in this article are solely those of the authors and do not necessarily represent those of their affiliated organizations, or those of the publisher, the editors and the reviewers. Any product that may be evaluated in this article, or claim that may be made by its manufacturer, is not guaranteed or endorsed by the publisher.

## Footnotes

1
https://ftp.ncbi.nlm.nih.gov/pub/taxonomy/

2
www.freebioinfo.org

## Supplementary Material

Supplementary Figure 1 Divergent responses of different genera as indicated by the log2 of ratio on the number of enriched (NEG) to diminished genes (NDG) were largely associated with the changes in gene abundance of each genus.
