# Supplementary figures and images for: Taxonomic and Functional Diversity of Rhizosphere Microbiome Recruited From Compost Synergistically Determined by Plant Species and Compost
Source: Front Microbiol. 2022 Jan 13;12:798476. doi: 10.3389/fmicb.2021.798476 (PMC8792965; doi:10.3389/fmicb.2021.798476)

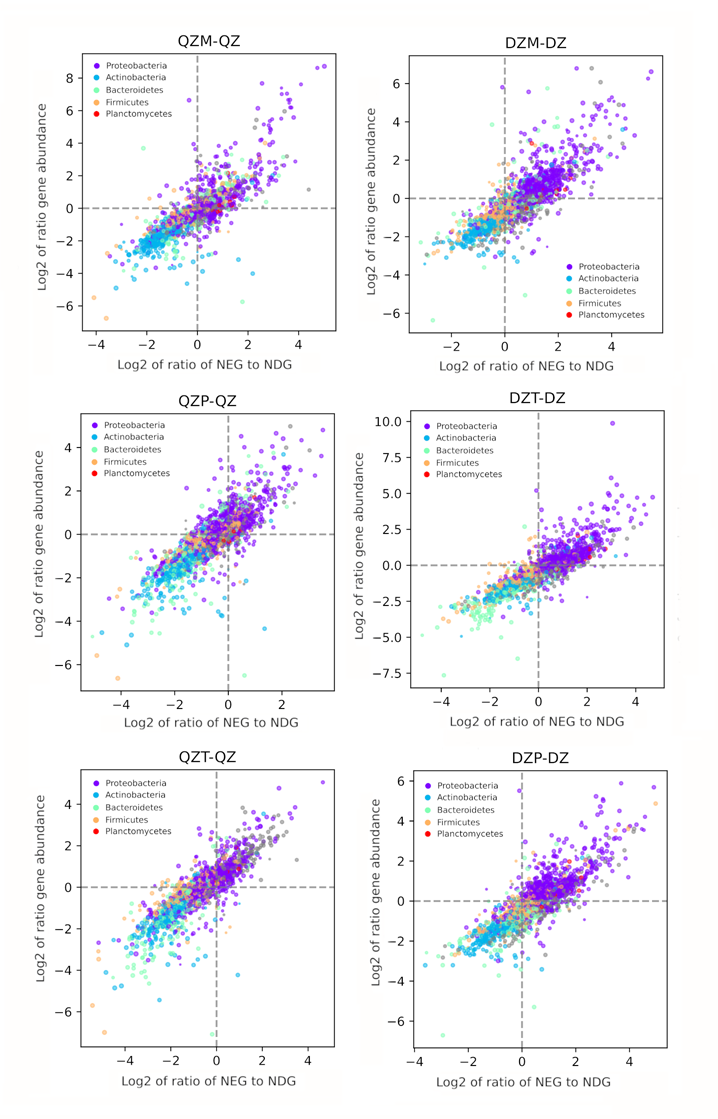

Supplement: Supplementary Figure 1 — Divergent responses of different genera as indicated by the log2 of ratio on the number of enriched (NEG) to diminished genes (NDG) were largely associated with the changes in gene abundance of each genus. [file Image_1.TIFF]
